# Immunocytokine augments local and abscopal response and animal survival when added to radiation and CTLA-4 checkpoint inhibition in a murine melanoma model

**DOI:** 10.1186/2051-1426-3-S2-P308

**Published:** 2015-11-04

**Authors:** Zachary S Morris, Emily I Guy, David M Francis, Monica M Gressett, Eric A Armstrong, Shyhmin Huang, Stephen D Gillies, Alan J Korman, Jacquelyn A Hank, Alexander L Rakhmilevich, Paul M Harari, Paul M Sondel

**Affiliations:** 1 Dept. of Human Oncology, University of Wisconsin School of Medicine and Public Health, Madison, WI, USA; 2 Provenance Biopharmaceuticals, Carlisle, MA, USA; 3 Bristol-Myers Squibb Company, Redwood City, CA, USA; 4 Department of Human Oncology, University of Wisconsin-Madison, Madison, WI, USA; 5 Department of Human Oncology, Department of Pediatrics, University of Wisconsin-Madison, Madison, WI, USA

## 

We have identified a cooperative interaction between radiation and intratumoral injection of anti-GD2 immunocytokine (hu14.18-IL2) in murine tumor models. In a moderate size (~200mm^3^), single tumor, B78 melanoma model this combination results in complete tumor regression in 71% of animals and a memory T cell response. We hypothesized that intratumoral immunocytokine would improve local and abscopal response to combined radiation and anti-CTLA-4 antibody.

Mice bearing large B78 tumors (~500mm^3^) were treated with single fraction (12Gy) or sham radiation, intratumoral immunocytokine or control IgG (50μg days 6-10 after radiation), and intraperitoneal IgG2a anti-CTLA-4 or control IgG (100μg days 3, 6, and 9 after radiation). In this large tumor model the effect of combined radiation and immunocytokine was reduced (27% complete response) and addition of immunocytokine to radiation and anti-CTLA-4 improved tumor response (73% complete response) and animal survival compared to doublet combinations of these agents. In a model of microscopic metastatic disease generated by IV injection of animals bearing large primary B78 tumors (~500mm^3^) with 4x10^5^ GD2-deficient B16 melanoma cells (parental to B78) on the day of radiation, we observed improved survival with the addition of immunocytokine to combined radiation and anti-CTLA-4.

However, in a model of established metastatic disease with a moderate size (~200mm^3^) primary B78 melanoma and a palpable (~50mm^3^) distant B78 tumor we did not observe an abscopal response when treating the primary tumor with radiation and intratumoral immunocytokine. Strikingly, when compared to animals with a single tumor we observed a profound inhibitory effect of the non-radiated second tumor such that primary tumor response to radiation and immunocytokine was indistinct from radiation alone in this two-tumor model. Delivering radiation to both the primary and secondary tumors eliminated this inhibitory effect of the secondary tumor. In this two-tumor model we combined primary tumor radiation and intratumoral immunocytokine with intraperitoneal IgG2b or IgG2a anti-CTLA-4. Both isotypes inhibit CTLA-4 activity but the latter has a reportedly greater ability to deplete intratumoral regulatory T cells (Tregs). While IgG2b anti-CTLA-4 had minimal effect on primary tumor response to radiation and immunocytokine, IgG2a anti-CTLA-4 rendered 80% of animals disease-free when given with radiation and immunocytokine, implicating Tregs in the suppressive effect of the second tumor on primary tumor response. In this two-tumor model, combination of radiation, immunocytokine, and IgG2a anti-CTLA-4 enhanced primary tumor and abscopal response as well as survival compared to doublet combinations. Clinical trial designs to explore these findings will be presented.

